# A comparison of two molecular methods for diagnosing leptospirosis from three different sample types in patients presenting with fever in Laos

**DOI:** 10.1016/j.cmi.2017.10.017

**Published:** 2018-09

**Authors:** K. Woods, C. Nic-Fhogartaigh, C. Arnold, L. Boutthasavong, W. Phuklia, C. Lim, A. Chanthongthip, S.M. Tulsiani, S.B. Craig, M.-A. Burns, S.L. Weier, V. Davong, S. Sihalath, D. Limmathurotsakul, D.A.B. Dance, N. Shetty, M. Zambon, P.N. Newton, S. Dittrich

**Affiliations:** 1) National Infection Service, Public Health England, London, UK; 2) Lao-Oxford-Mahosot Hospital-Wellcome Trust Research Unit, Microbiology Laboratory, Mahosot Hospital, Vientiane, Laos; 3) Bart's Health Division of Infection, Pathology and Pharmacy Department, Royal London Hospital, London, UK; 4) Mahidol-Oxford-Research Unit, Faculty of Tropical Medicine, Mahidol University, Bangkok, Thailand; 5) Queensland Health Forensic and Scientific Service, WHO Collaborating Centre for Reference and Research on Leptospirosis, Brisbane, Qld, Australia; 6) University of the Sunshine Coast, Faculty of Science Health, Education and Engineering, Sippy Downs, Qld, Australia; 7) Faculty of Health, Queensland University of Technology, Brisbane, Qld, Australia; 8) Centre for Tropical Medicine and Global Health, Nuffield Department of Medicine, University of Oxford, Oxford, England, UK; 9) Faculty of Infectious and Tropical Diseases, London School of Hygiene and Tropical Medicine, London, UK; 10) Foundation for Innovative New Diagnostics, Geneva, Switzerland

**Keywords:** Bayesian, Buffy coat, Laos, Latent class model, *Leptospira*, Leptospirosis, Molecular diagnosis, Quantitative PCR, Serum, Urine

## Abstract

**Objectives:**

To compare two molecular assays (*rrs* quantitative PCR (qPCR) versus a combined 16SrRNA and *LipL32* qPCR) on different sample types for diagnosing leptospirosis in febrile patients presenting to Mahosot Hospital, Vientiane, Laos.

**Methods:**

Serum, buffy coat and urine samples were collected on admission, and follow-up serum ∼10 days later. *Leptospira* spp. culture and microscopic agglutination tests (MAT) were performed as reference standards. Bayesian latent class modelling was performed to estimate sensitivity and specificity of each diagnostic test.

**Results:**

In all, 787 patients were included in the analysis: 4/787 (0.5%) were *Leptospira* culture positive, 30/787 (3.8%) were MAT positive, 76/787 (9.7%) were *rrs* qPCR positive and 20/787 (2.5%) were 16SrRNA/*LipL32* qPCR positive for pathogenic *Leptospira* spp. in at least one sample. Estimated sensitivity and specificity (with 95% CI) of 16SrRNA/*LipL32* qPCR on serum (53.9% (33.3%–81.8%); 99.6% (99.2%–100%)), buffy coat (58.8% (34.4%–90.9%); 99.9% (99.6%–100%)) and urine samples (45.0% (27.0%–66.7%); 99.6% (99.3%–100%)) were comparable with those of *rrs* qPCR, except specificity of 16SrRNA/*LipL32* qPCR on urine samples was significantly higher (99.6% (99.3%–100%) vs. 92.5% (92.3%–92.8%), p <0.001). Sensitivities of MAT (16% (95% CI 6.3%–29.4%)) and culture (25% (95% CI 13.3%–44.4%)) were low. Mean positive Cq values showed that buffy coat samples were more frequently inhibitory to qPCR than either serum or urine (p <0.001).

**Conclusions:**

Serum and urine are better samples for qPCR than buffy coat, and 16SrRNA/*LipL32* qPCR performs better than *rrs* qPCR on urine. Quantitative PCR on admission is a reliable rapid diagnostic tool, performing better than MAT or culture, with significant implications for clinical and epidemiological investigations of this global neglected disease.

## Introduction

Leptospirosis is a leading cause of morbidity and mortality globally with an estimated 1 million cases and 60 000 deaths annually [1]. In South East Asia there are an estimated 55.5 cases per 100 000 annually, with an estimated mortality of 2.96/100 000 [1]. In temperate regions, leptospirosis is the third commonest infectious cause of life-threatening disease in returning travellers [2].

Leptospirosis presents as a non-specific febrile illness that can progress to serious complications [3], [4], [5] with up to 40% mortality if untreated [6]. Diagnosis is often delayed as *Leptospira* species grow slowly in culture, and the reference standard Microscopic Agglutination Test (MAT) requires acute and convalescent sera, making diagnosis retrospective by nature. Culture and MAT are therefore poor clinical diagnostic tools for leptospirosis. Furthermore, they are imperfect reference standards, necessitating the use of statistical models such as the Bayesian latent class model to determine the true accuracy of alternative *Leptospira* diagnostics [7], [8], [9].

Several molecular assays for *Leptospira* spp. have been developed, targeting housekeeping genes such as *gyrB* [10], *rrs* (16SrRNA) [11] and *secY* [12], or pathogen-specific *LipL32* [13], *ligA* and *ligB* [14], which avoid amplification of non-pathogenic *Leptospira* species. Large-scale prospective evaluations in endemic tropical settings are lacking and uncertainty remains regarding the optimum sample for molecular detection of *Leptospira* spp. with buffy coat [13], [15], serum [16] and urine [13], [17] all recommended.

We prospectively evaluated the *rrs* quantitative PCR (qPCR) [18] alongside an assay for 16SrRNA and *LipL32* developed by Public Health England (henceforth 16SrRNA/*LipL32* qPCR) using admission serum, buffy coat (BC) and urine samples from febrile patients presenting to Mahosot Hospital, Vientiane, Laos.

## Materials and methods

### Retrospective study

The 16SrRNA/*LipL32* qPCR was evaluated using stored (–80°C) admission serum and BC samples from 59 cases of leptospirosis (positive by: culture *n* = 19; MAT *n* = 20 (admission titre ≥1:400 or four-fold convalescent rise); or *rrs* qPCR on BC *n* = 20) and 83 controls (diagnoses identified in a published study [19], see Supplementary material, Table S1). Frozen DNA previously extracted from BC was used in 43/59 cases and all 83 controls, because stored samples were not available for fresh extraction.

### Prospective study

#### Study population

A total of 1471 consecutive patients presented with a febrile illness to Mahosot Hospital between 30 May and 30 November 2014, of which 811 were included. Inclusion criteria were: fever (history of fever or documented temperature ≥38°C), plus at least one of: headache, rash, myalgia, arthralgia, lymphadenopathy, meningitis, encephalitis, respiratory symptoms, jaundice, or acute renal failure. Exclusion criteria were: age <6 months; fever duration >1 month; admission diagnosis of: wound infection; diabetic foot infection; postoperative infection; abscess; parotitis; urine infection; or diarrhoea. All participants (or their parents/guardians) provided written informed consent before sample collection. Ethical approval for all investigations was granted by the Oxford Tropical Research Ethics Committee (University of Oxford, UK) and the National Ethics Committee for Health Research, Lao PDR.

#### Sample processing

Samples were collected at presentation from the 811 patients: serum (*n* = 785), EDTA BC (*n =* 774), blood clot (*n* = 811) and urine (*n* = 644). The BC were obtained by centrifuging EDTA blood at 3200 ***g*** for 8 min. Convalescent serum was collected 10–14 days later when possible (*n* = 248). Samples were stored at +4°C until DNA preparation.

##### DNA preparation

The 1.5-mL urine aliquots were centrifuged at 20 000 ***g***, retaining the pellet with 200 μL urine for DNA extraction. Manual DNA extraction was performed on BC, serum and urine using the QIAamp DNA Minikit (Qiagen, Hilden, Germany) within 7 days of sampling [19]. Ten microlitres of GFP-plasmid *Escherichia coli* control (10^8^/mL) was added to each sample before extraction as a process and inhibition control.

##### Molecular detection

The 16SrRNA/*LipL32* qPCR includes two reaction mixes per sample: a duplex assay targeting *LipL32* and an internal control (GFP *E. coli* plasmid), and a triplex assay targeting the 16SrRNA gene. The triplex assay probes correlate with genomic variants of pathogenic, intermediate and environmental *Leptospira* strains (see Supplementary material, Fig. S1). Comparison of cycle threshold (Cq) values for these probes distinguishes pathogenic from non-pathogenic *Leptospira* spp. (Public Health England, unpublished data; see Supplementary material, Table S2). Quantitative PCRs were performed with 5 μL DNA. The *rrs* qPCR was performed as described previously [18]. Each of the 20-μL 16SrRNA and *LipL32* qPCR reaction mixes contained: 12.5 μL Fast Bluex2 Master Mix (Eurogentec, Southampton, UK), 0.5 μm of each primer and 0.125 μm of each probe. Cycling conditions were: 95°C for 5 min, then 50 cycles of: 95°C for 3 s, 60°C for 30 s, 72°C for 10 s. Each qPCR run included standard curves (∼1 genome equivalent (GE)/μL – 10^3^ GE/μL; Lao clinical isolate, assumed genome size ∼4.7 Mb) and non-template controls (which were always negative). The qPCRs were performed in weekly batches using a Rotorgene 6000 (Qiagen) or CFX96 Touch (Bio-Rad Laboratories Ltd, Hercules, CA). Separate investigators (blinded to clinical data and other results) performed the 16SrRNA/*LipL32* qPCR (KW) and the *rrs* qPCR (WP).

##### Culture

Blood clots were cultured for *Leptospira* spp. (as previously described [20]). by investigators blinded to the qPCR results.

##### Serology

MAT was performed at the WHO/FAO/OIE Collaborating Centre for Leptospirosis Reference and Research, Queensland, Australia (see Supplementary material, Table S3). Criteria for a confirmed leptospirosis diagnosis were a single MAT titre of ≥1:400 or a four-fold convalescent rise in titre [21].

#### Data analysis

##### Result interpretation

The *rrs* qPCR was considered positive with a Cq ≤40 [22]. The 16SrRNA/*LipL32* qPCR was considered positive with a Cq ≤45 and GFP internal control Cq≤35 (see Supplementary material, Table S2). If interpretation of the 16SrRNA/*LipL32* qPCR was equivocal despite a GFP Cq within the normal range, then the 16SrRNA/*LipL32* qPCR was repeated in triplicate to obtain the final result. Only 16SrRNA/*LipL32* qPCR results indicating the detection of pathogenic *Leptospira* DNA were considered positive for the comparative analysis with the *rrs* qPCR.

##### Diagnostic characteristics

Sensitivity and specificity of the *rrs* and 16SrRNA/*LipL32* qPCR for diagnosing leptospirosis were calculated using MAT or culture positive as the combined reference standard. McNemar's exact test was used for statistical comparisons. Bayesian Latent Class Modelling (LCM) was performed using WinBUGS 1.4 software [23] to estimate the true accuracy of each diagnostic test as described previously [7], [8], [9] (Table 3). Mean positive Cq values were also compared for *rrs* and 16SrRNA/*LipL32* qPCR when pathogenic *Leptospira* DNA was detected and for sample type. Mean GFP Cq was calculated with Cq = 50 for samples with no GFP Cq. Basic statistical assessments were done using STATA (Stata/MP 14.1 for Mac, College Station, TX, USA).

## Results

### Retrospective study

There was no significant difference in performance between *rrs and* 16SrRNA/*LipL32* qPCR for diagnosing cases or controls or between sample types (see Supplementary material, Table S4); nor in Cq values between serum and BC for *rrs* (p 0.86) or 16SrRNA/*LipL32* (p 0.44) qPCR.

### Prospective study

**Fig. 1:**
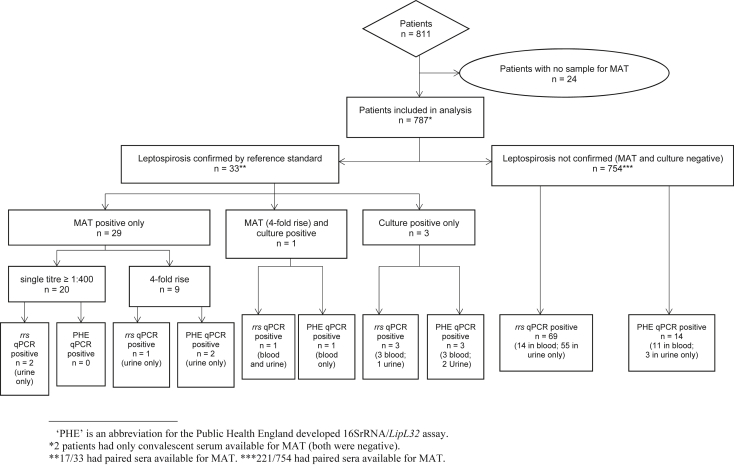
Participant flow and diagnostic test results.

Twenty-four of 811 patients did not have serum available for reference testing (MAT) and were excluded from analysis (Fig. 1). Sample types available for qPCR varied (see Supplementary material, Fig. S2). Only 238 (30.2%) patients had paired sera available for MAT testing, of whom 221 were negative by the combined reference standard of MAT and culture. Convalescent serum samples were taken a median of 10 days after admission (interquartile range 7–14 days). Median patient age was 39 years (range 0.5–97 years), 58% were male. Median duration of fever at presentation was 5 days (interquartile range 3–7 days; range 1–30 days).

Seventy-six patients (9.7%) were *rrs* qPCR positive, 58 in urine only (7.4%). 16SrRNA/*LipL32* qPCR detected pathogenic *Leptospira* DNA in 20 patients (2.5%; Fig. 1) with no sample positive by *LipL32* qPCR alone, and intermediate *Leptospira* DNA detected in an additional 30 patients (3.8%; serum = 12, BC = 4, urine = 14). The combination of sample types that were qPCR positive in each patient varied (see Supplementary material, Fig. S3). In addition, concordance of *rrs* and 16SrRNA/*LipL32* qPCRs in patients positive for pathogenic *Leptospira* DNA was low: serum 52.9% (9/17), BC 46.1% (6/13) and urine only 8.8% (6/68) were positive by both qPCRs.

#### Clinical characteristics of pathogenic *Leptospira* spp.-positive patients

The median age of 33 patients positive by MAT (*n* = 30) or culture (*n* = 4) was 35 (range 8–75) years, 76% were male (25/33) and four died (12%) in hospital. Of 74 patients who were leptospirosis positive only by qPCR, three died (4%). Median fever duration at admission was significantly shorter for patients positive for pathogenic *Leptospira* spp. by any qPCR in blood than by MAT (3.5 versus 7 days, p <0.001) (Table 1). Mortality analysis was limited by incomplete data, but was not significantly higher in patients who were qPCR positive in blood on admission than MAT-positive patients (p 0.43).

**Table 1:** Median fever duration and mortality for patients positive for leptospirosis by the different tests

	Leptospirosis positive by:							
	MAT *n* = 30	Culture *n* = 4	Any PCR (*n* = 82)					
			Blood only (*n* = 14)		Urine only (*n* = 61)		Blood and urine (*n* = 7)	
			*rrs* (*n* = 12^a^)	PHE (*n* = 10^b^)	*rrs* (*n* = 58)	PHE (*n* = 5)	*rrs* (*n* = 6)	PHE (*n* = 5)
Median fever days on admission (IQR)	7 (5–14)	4 (3–5.5)	3.5 (2.5–7)	3 (2–4)	6 (3–10)	7 (6–7)	3 (2–4)	4 (3–4)
Mortality	12.5% (2/16^c^)	50% (2/4)	25% (3/12)	10% (1/10)	n/a^c^ (0/5)	0% (0/5)	33.3% (2/6)	60% (3/5)

PHE = 16SrRNA/LipL32.

a Three patients had no urine sample for testing.

b Four patients had no urine sample for testing.

c Mortality data for 14 MAT-positive patients and 53 patients who were only positive by *rrs* qPCR on urine was not available.

**Fig. 2:**
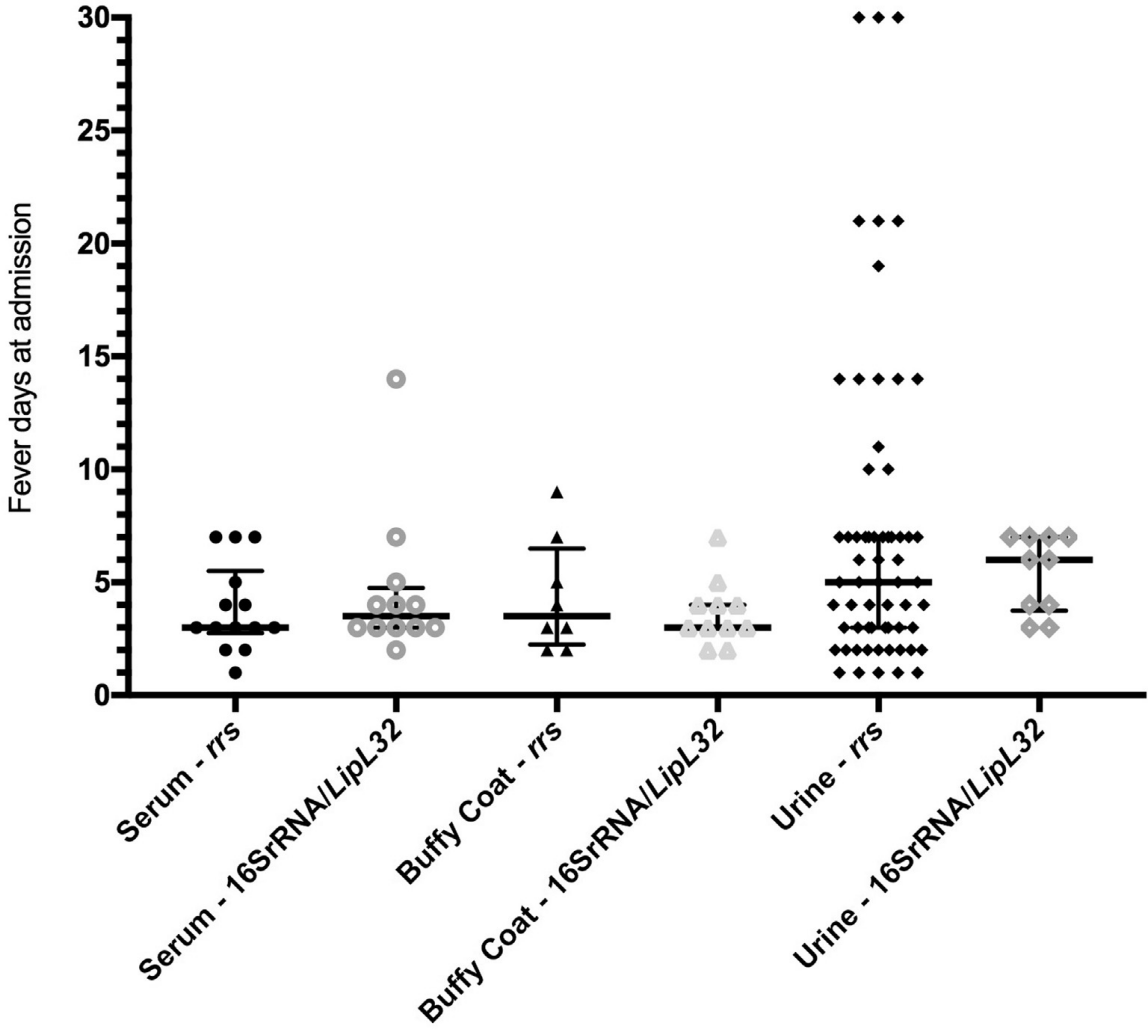
Fever duration at admission in patients qPCR positive for pathogenic Leptospira DNA, according to admission sample type which was qPCR positive.

There was no significant difference between median fever duration at admission across sample types for *rrs* (p 0.2) or 16SrRNA/*LipL32* (p 0.08) qPCR (Fig. 2). Most qPCR-positive urine samples were within 7 days of fever onset.

#### Diagnostic accuracy

Compared with the reference standard, sensitivities of both qPCRs were <20% for all sample types, with no significant difference between *rrs* and 16SrRNA/*LipL32* assays (serum p >0.99, BC p 0.08, urine p 0.65; Table 2). Specificities were ≥98.5% for all samples, except that *rrs* qPCR was significantly less specific on urine compared with 16SrRNA/*LipL32* qPCR (90% versus 99%, p <0.001).

**Table 2:** Conventional analysis of diagnostic accuracy using positivity of MAT or culture (*n* = 33) as the reference standard

Sample type	PCR	Reference standard		Specificity % (95% CI)	Sensitivity % (95% CI)
		Positive (*n* = 33)	Negative (*n* = 754)		
Serum (*n* = 766)	*rrs*	3	11^a^	9.38 (1.98–25.0)	98.5 (97.3–99.3)
	PHE	3	9^a^	9.38 (1.98–25.0)	98.8 (97.7–99.4)
Buffy coat (*n* = 750)	*rrs*	1	7^a^	3.03 (0.08–15.8)	99.0 (98.0–99.6)
	PHE	4	7^a^	12.1 (3.4–28.2)	99.0 (98.0–99.6)
Urine (*n* = 626)	*rrs*	5	59^a^	17.2 (5.85–35.8)	90.1 (87.4–92.4)
	PHE	4	6^a^	13.8 (3.89–31.7)	99.0 (97.8–99.6)

a Number of samples negative by the reference standard but positive by PCR that had paired MAT samples: serum: rrs (*n* = 6), PHE (*n* = 5); buffy coat: rrs (*n* = 5), PHE (*n* = 3); urine: rrs (*n* = 23), PHE (*n* = 1).

**Table 3:** Bayesian Latent Class Modelling estimates of diagnostic accuracy for each test

Parameters	Bayesian LCM% (95% credibility interval)
Prevalence	2.0 (1.1–3.8)
MAT	
Sensitivity	15.8 (6.3–29.4)
Specificity	96.5 (96.2–96.9)
PPV	10.0 (3.3–20.0)
NPV	98.3 (96.7–98.9)
Culture for *Leptospira* spp.	
Sensitivity	25.0 (13.3–44.4)
Specificity	100
PPV	100
NPV	98.5 (96.7–99.4)
16SrRNA/*LipL32* qPCR on serum	
Sensitivity	53.9 (33.3–81.8)
Specificity	99.6 (99.2–100)
PPV	75.0 (50.0–100)
NPV	99.1 (97.6–99.7)
16SrRNA/*LipL32* qPCR on buffy coat	
Sensitivity	58.8 (34.4–90.9)
Specificity	99.9 (99.6–100)
PPV	90.9 (72.7–100)
NPV	99.1 (97.4–99.9)
16SrRNA/*LipL32* qPCR on urine	
Sensitivity	45.0 (27.0–66.7)
Specificity	99.6 (99.3–100)
PPV	70.0 (50.0–100)
NPV	98.8 (97.3–99.5)
*rrs* qPCR on serum	
Sensitivity	50.0 (29.6–77.8)
Specificity	99.2 (99.0–99.5)
PPV	57.1 (42.9–71.4)
NPV	99.0 (97.3–99.7)
*rrs* qPCR on buffy coat	
Sensitivity	35.7 (20.7–55.6)
Specificity	99.7 (99.5–100)
PPV	75.0 (50.0–100)
NPV	98.7 (97.1–99.5)
*rrs* qPCR on urine	
Sensitivity	39.1 (25.0–57.1)
Specificity	92.5 (92.3–92.8)
PPV	9.4 (6.3–14.1)
NPV	98.6 (97.0–99.5)

Note: Culture specificity was fixed at 100%. The Akaike Information Criterion was used to evaluate goodness of fit and select the final model. The final Bayesian Latent Class Modelling (LCM) included culture, MAT, 16SrRNA/*LipL32* qPCR on serum and urine samples, and *rrs* qPCR on buffy coat samples with conditional dependence between culture and qPCR assays on blood samples (see Supplementary material, Table S5). Sensitivity and specificity of all tests and Bayesian p-values were estimated (see Supplementary material, Table S6).

Bayesian LCM estimates of unbiased sensitivities of all qPCRs were higher than those estimated by conventional analysis in all sample types, and higher than MAT or culture (Table 3). Estimated unbiased specificities of all qPCRs were similar to those derived from conventional analyses. There was no significant difference in sensitivity between the qPCR assays on serum (Bayesian p 0.082) or urine (Bayesian p 0.092) samples. On BC samples 16SrRNA/*LipL32* qPCR sensitivity was higher than *rrs* qPCR (Bayesian p <0.001).

Sensitivity analysis including only patients with all three sample types available for qPCR testing (*n* = 597) obtained similar results (see Supplementary material, Tables S7 and S8).

#### Sample type comparison

Mean Cq value did not differ significantly between the three sample types for detection of pathogenic *Leptospira* spp. with *rrs* (p 0.69) or 16SrRNA/*LipL32* (16S p 0.19; LipL32: p 0.46*)* qPCRs. However, BC samples were significantly more frequently inhibitory (48/750, 6.4%) than serum (6/766, 0.78%) or urine (8/626,1.3%) (p <0.001).

Five patients had pathogenic *Leptospira* DNA detected by 16SrRNA/*LipL32* qPCR in urine but not in blood (see Supplementary material, Fig. S3); two of these had paired sera available for MAT, which confirmed leptospirosis by a four-fold titre rise. Of the 58 patients with *rrs* qPCR positive in urine but not blood, only three were confirmed by MAT (23/58 had paired sera available). Thirteen of the 55 patients not MAT confirmed were also positive by 16SrRNA/*LipL32* qPCR in urine (one pathogenic, one intermediate and 11 non-pathogenic *Leptospira* DNA).

## Discussion

We compared two molecular assays and three different sample types for diagnosing acute leptospirosis in Laos. Performance of the qPCRs was similar and consistent with previous reports [7], [18], [24] with high specificity but only 40%–60% sensitivity when Bayesian LCM was used to estimate the unbiased accuracy of each test. Pre-hospital antibiotic use may contribute to low qPCR sensitivity in our population, with detectable antibiotic activity found in urine of 57% of febrile patients presenting to Mahosot Hospital [25]. Duration of leptospiraemia also affects qPCR sensitivity and, as expected, samples collected after the first week of illness were rarely qPCR positive in this study. Nevertheless, molecular detection from admission blood identified 17 additional *Leptospira* infections compared with the conventional reference standard MAT. Our findings are consistent with the previous meta-analysis showing that culture and MAT have low sensitivities [7]. Low sensitivity of MAT and culture were also supported by post-hoc estimation of sensitivities among patients with pathogenic *Leptospira* DNA-positive qPCR in blood and paired sera available for MAT (see Supplementary material, Table S9). In addition, our study suggests that MAT has imperfect specificity in our setting (96.5%), possibly related to frequent *Leptospira* spp. exposure confounding interpretation of this serological test in acutely febrile patients in Laos.

The stated limit of detection for both *rrs* and 16SrRNA/*LipL32* qPCR is one genome copy per reaction [18] (PHE, unpublished data) and similar performance of the two assays was therefore expected. However, the *rrs* and 16SrRNA/*LipL32* assays target different sections of the 16SrRNA gene, which may explain some of the observed assay discordance. Before this study, BC was routinely used for molecular detection of *Leptospira* spp. in Laos [3], [19], in line with the hypothesis that phagocytosed *Leptospira* spp. are concentrated in BC. However, this study found no difference in sensitivity between serum and BC for qPCR diagnosis of leptospirosis and identified serum as a better blood matrix than BC due to the significantly lower inhibition rate with serum samples. This is consistent with previous reports of qPCR inhibition with BC [26], and use of BC for qPCR may have resulted in underestimation of leptospirosis frequency in previous studies [19].

In line with previous findings [27], qPCR inhibition was rare with urine samples in our study and, with no difference in sensitivity to blood, urine is a useful sample for the molecular diagnosis of leptospirosis, particularly when using the more specific 16SrRNA/*LipL32* qPCR. Detection of intermediate or non-pathogenic *Leptospira* strains in urine by *rrs* qPCR, although previously reported [18], does not fully explain the lower specificity of *rrs* qPCR on urine as only 22% of urine samples positive by *rrs* qPCR alone had intermediate or non-pathogenic *Leptospira* DNA detected by 16SrRNA/*LipL32* qPCR. Although *rrs* qPCR analytical specificity has been shown to be high [11], [18], a recent prospective study [28] identified false-positive results of *rrs* qPCR on blood culture fluid containing non-leptospiral bacteria. Urine is more likely than blood to contain contaminating bacteria and it is possible that this accounts for the apparent high false-positive rate of *rrs* qPCR on urine in our study. Environmental contamination of urine samples was minimized in our study by the use of sterile containers and clear instructions for sample collection. Although the timing of *Leptospira* excretion in urine in humans is not clearly defined, our data support the findings of Iwasaki et al. [29] that *Leptospira* DNA detection by qPCR in urine occurs both early and late in the acute phase of leptospirosis.

A recent study in Ecuador found that intermediate *Leptospira* strains might contribute more to human leptospirosis than previously believed [30], a finding that our data seem to support with 1.5 times more patients positive for intermediate *Leptospira* spp. than pathogenic *Leptospira* spp. by 16SrRNA/*LipL32* qPCR. Distinguishing pathogenic from intermediate and non-pathogenic strains of *Leptospira* species is an advantage of the 16SrRNA/*LipL32* qPCR for furthering our understanding of the role of these species in human leptospirosis. However, the complexity of the assay is a significant limitation for deployment to resource-limited settings where leptospirosis is most prevalent. The simpler *rrs* assay used with the optimum sample type (serum) represents a workable alternative.

A limitation of our study was the unexpectedly low prevalence of leptospirosis, resulting in low positivity rates across all tests and wide 95% credible intervals for the diagnostic accuracy values. However, only such prospective studies can determine the true utility of diagnostic tests and optimum samples in routine practice. Additional limitations include the low proportion of patients with paired sera available for MATs, use of blood clot for *Leptospira* culture [20], that only three-quarters of patients had all sample types available for qPCR, and limited outcome data. These reflect the difficulty of specimen collection in clinical settings, particularly in low- and middle-income countries.

In conclusion, molecular diagnostics are important for accurate and timely diagnosis of leptospirosis with qPCR performing consistently better than culture or MAT, and our data demonstrate the importance of Bayesian LCM for assessing diagnostic tests when reference standards are imperfect [7], [9]. We identified serum as the most suitable sample overall for qPCR. Our data highlight the challenges associated with *Leptospira* diagnostics and the need for product development and evaluation to ensure that rapid, reliable diagnostics are available to guide patient management and reduce leptospirosis morbidity and mortality globally.

## Transparency declaration

None of the authors have any conflicts of interest to declare.

## Funding

This study was funded by the Wellcome Trust of Great Britain (grant nos.: 106698/Z/14/Z and 106698/B/14/Z). The secondment of authors KW and CNF to Laos was funded by Public Health England (RPW1).

## Appendix A Supplementary data

**Table S1** Diagnoses of control patients in retrospective study.

**Table S2** 16SrRNA/*LipL32* quantitative PCR interpretation.

**Table S3** Microscopic agglutination test serovar panel.

**Table S4** Retrospective study conventional analysis of diagnostic accuracy.

**Table S5** Bayesian Latent Class Models data set (*n* = 787).

**Table S6** Bayesian Latent Class Models WinBUGS code.

**Table S7** Bayesian Latent Class Model outputs for patients with all three of serum, buffy coat and urine sample types available for quantitative PCR (*n* = 597).

**Table S8** Bayesian Latent Class Model data set for those with all three of serum, buffy coat and urine samples available for quantitative PCR (*n* = 597).

**Table S9** Naive sensitivities of MAT and culture in patients quantitative PCR positive for pathogenic *Leptospira* spp. in blood and with paired sera available for microscopic agglutination test (*n* = 238).

**Fig. 1** 16SrRNA/*LipL32* quantitative PCR primer and probe sequences and alignments.

**Fig. 2** Sample types available from patients included in analysis.

**Fig. 3** Combination of sample types quantitative PCR positive in patients with detectable pathogenic *Leptospira* DNA in at least one sample.
