# Supplementary material for: A comparison of two molecular methods for diagnosing leptospirosis from three different sample types in patients presenting with fever in Laos
Source: Clin Microbiol Infect. 2018 Sep;24(9):1017.e1–7. doi: 10.1016/j.cmi.2017.10.017 (PMC6125144; doi:10.1016/j.cmi.2017.10.017)
Supplement: Supplementary file 2 [file mmc2.docx]

Supplementary Figure 1a. Positions and sequences of the LipL32 probe and primers

10 20 30 40 50 60 70 80 90 100 110 120 130 140 150

....|....|....|....|....|....|....|....|....|....|....|....|....|....|....|....|....|....|....|....|....|....|....|....|....|....|....|....|....|....|

**Interrogans Consensus**  **GAAAAAACTTTCGATTHTGGCHATCTCCGTTGCACTCTTTGCAAGCATTACCGCTTGTGGTGCBTTCGGTGGTCTGCCAAGCCTAAAAAGCTCTTTTGTTCTGAGCGAGGASACAATCCCAGGGACAAACGAAACCGTAAAAACGTTACT**

**Noguchii Consensus**  **......................................................................................................................................................**

**Kirschneri Consensus**  **......................................................................................................................................................**

**Santarosai Consensus**  **....................................................................................T........................AG....G.T..G..A.......................C..**

**Weilii Consensus**  **.....................G.......Y...........................................T..........T........................AG....G.T.....A.......................C..**

**Borgpetersenii Consensus** **.....................K.......Y.................................K.........Y..........W........................RR....R.Y.....R.......................M..**

160 170 180 190 200 210 220 230 240 250 260 270 280 290 300

....|....|....|....|....|....|....|....|....|....|....|....|....|....|....|....|....|....|....|....|....|....|....|....|....|....|....|....|....|....|

**Interrogans Consensus**  **TCCCTACGGATCTGTGATCAACTATTACGGATACGTAAAGCCAGGACAAGCGCCGGACGGTTTAGTCGATGGAAACAAAAAAGCATACTATCTCTATGTTTGGATTCCTGCCGTAATCGCTGCAAATGGGAGTTCGTATGATTTCCCCAA**

**Noguchii Consensus**  **..........................................................................................................................-...........................**

**Kirschneri Consensus**  **..........................................................................................................................-...........................**

**Santarosai Consensus**  **......T..........................T.......................T..C........C......................................A..T..........-...........................**

**Weilii Consensus**  **......T..........................T...................................C...................................C..A..T..........-...........................**

**Borgpetersenii Consensus** **......Y..........................Y...................................Y...................................Y..W..Y..........-...........................**

**LipL32AF GGATCTGTGATCAACTATTACGGATA> TAAAGCCAGGACAAGCGCCG <CATTAGCGACGTTTACCC LipL32AR**

(62.8°C) FAM BHQ1 (61.6°C)

LipL32AProbe(71.3°C) - Amplicon 123 bp

310 320 330 340 350 360 370 380 390 400 410 420 430 440 450

....|....|....|....|....|....|....|....|....|....|....|....|....|....|....|....|....|....|....|....|....|....|....|....|....|....|....|....|....|....|

**Interrogans Consensus**  **CAGGCGAAATCGGTGARCCAGGCGAYGGAGACTTAGTAAGCGACGCTTTCAAAGCGGCTACCCCAGAAGAAAAATCAATGCCACATTGGTTTGATACTTGGATCCGTGTAGAAAGAATGTCGGCGATTATGCCTGACCAAATCGYCAAAG**

**Noguchii Consensus**  **..........................................................W..............................................................K.......................M....**

**Kirschneri Consensus**  **......................................................................................................................................................**

**Santarosai Consensus**  **...............................T........T..T..G...........A..................................................T..............A........C...........T....**

**Weilii Consensus**  **.............G.............................Y..............A.....G................................C....................................................**

**Borgpetersenii Consensus** **.............K.............................Y..............W.....R................................Y....................................................**

The figure shows the positions of the forward and reverse primers (LipL32AF and LipL32AR) and the probe LipL32A.

Supplementary Figure 1b. Positions and sequences of the 16S probes and primers

310 320 330 340 350 360 370 380 390 400 410 420 430 440 450

....|....|....|....|....|....|....|....|....|....|....|....|....|....|....|....|....|....|....|....|....|....|....|....|....|....|....|....|....|....|

**Interrogans Consensus**  **GCTCACCAAGGCGACGATCGGTAGCCGGCCTGAGAGGGTGTTCGGCCACAATGGAACTGAGACACGGTCCATACTCCTACGGGAGGCAGCAGTTAAGAATCTTGCTCAATGGGGGGGAACCCTGAA-GCAGCGACGCCGCGTGAACGATG**

**Alexanderi Consensus**  **....................................................................................................................-.........-.......................**

**Borgpetersenii Consensus** **..............................................................................................................................A.......................**

**Kirschneri Consensus**  **....................................................................................................................-.........-.......................**

**Kmetyi**  **....................................................................................................................-.........-.......................**

**Noguchii Consensus**  **...Y................................................................................................................-.........-.......................**

**Santarosai Consensus**  **.......................R............................................................................................-.........-.......................**

**Weilii Consensus**  **....................................................................................................................-.........-.......................**

**Genomosp 1**  **....................................................................................................................-.........-.......................**

**Broomii Consensus**  **.........................................C.........................................................................A-.........-.....................A.**

**Fainei Consensus**  **.........................................C.........................................................................A-.........-.....................A.**

**Inadai Consensus**  **.........................................C.........................................................................A-.........-.....................A.**

**Licerasiae Consensus**  **.........................................C.......................................................................C.C-..G......-.....................A.**

**Biflexa Consensus**  **..YY...............A...R................AA..........................SS...........................................C.A-..G......-.......................**

**Meyeri Consensus**  **..CT...............A....................AA.......................................................................C.A-..G......-.......................**

**Wolbachii Consensus**  **..Y................A....................AA.......................................................................C.A-..G......-.......................**

**Genomosp 4**  **..C................A....................AA.......................................................................C.A-..G......-.......................**

**Genomosp 3**  **...................A....................AA.......................................................................C.A-..G......-.......................**

**Genomosp 5**  **..CT...............A....................AA.......................................................................C.A-..G......-.......................**

**Borrelia**  **..CT..........T...AA...A................AA...T....C....................G....................C..........C.G.......C.A-..G.....C-.G......A.T.......T..A.**

**Treponema**  **............A.....A.T..T.......A........AA...A....T...G.......T....C...G....................C.......A..C.G......A..A-...T....C-.G..............G.T....**

LeptoF1 (61.04) GATCGGTARCCGGCCT>AGAGGGTG**TT**CGGCCACAATG PathProbe (70.35) <AATTCTTAGAACGAGTTACCC LeptoR (60.59)

FAM BHQ1 amplicon 98-99 bp

AGAGGGTG**TC**CGGCCACAAT InterProbe (70.31)

YY/JOE BHQ1

LeptoF2 (61.23) CGATCAGTARCCGGCCT>AGAGGGTG**AA**CGGCCACAATG EnviroProbe (70.3)

CY5 BHQ2

The figure shows the positions of the forward and reverse primers (LeptoF1, LeptoF2 and LeptoR) and the three probes (Path, Inter and Enviro) with key sequence differences highlighted in bold.

Supplementary Figure 1c. GFP *E. coli* plasmid control primer and probe sequences (5’ to 3’; included in the LipL32 reaction mix).

Forward Primer: CCTGTCCTTTTACCAGACAACCA

Reverse Primer: GGTCTCTCTTTTCGTTGGGATC

Probe: Cy5-TACCTGTCCACACAATCTGCCCTTTCG-BHQ2.
