# Supplementary material for: A comparison of two molecular methods for diagnosing leptospirosis from three different sample types in patients presenting with fever in Laos
Source: Clin Microbiol Infect. 2018 Sep;24(9):1017.e1–7. doi: 10.1016/j.cmi.2017.10.017 (PMC6125144; doi:10.1016/j.cmi.2017.10.017)
Supplement: Supplementary file 3 [file mmc3.docx]

**Supplementary Figure 2. Combination of sample types available from each patient (n=787) included in the final analysis** (Venn diagrams are not proportional)**.**


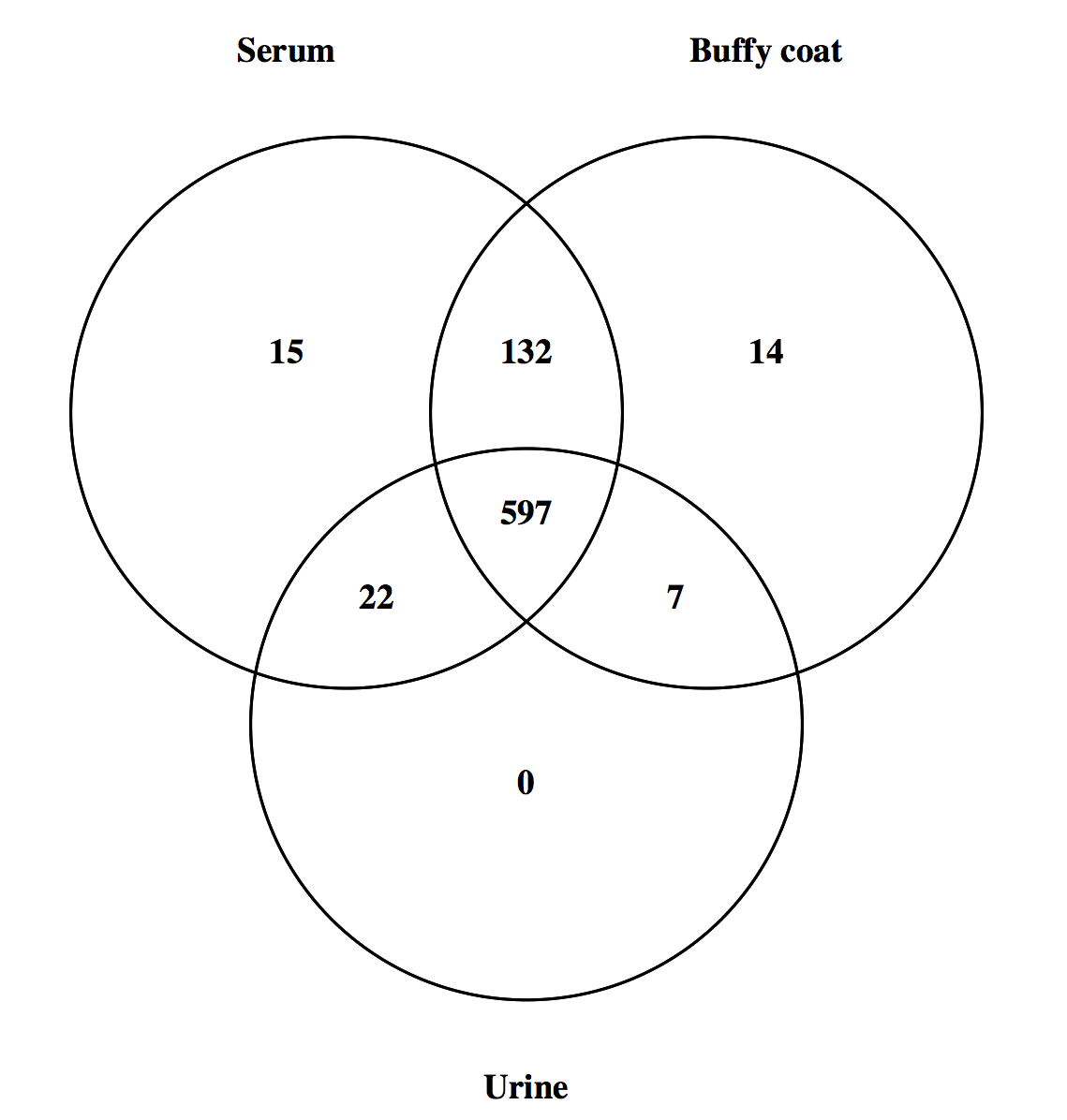


**Supplementary Figure 3. Patients according to sample type positive by qPCR** (Venn diagrams not proportional)


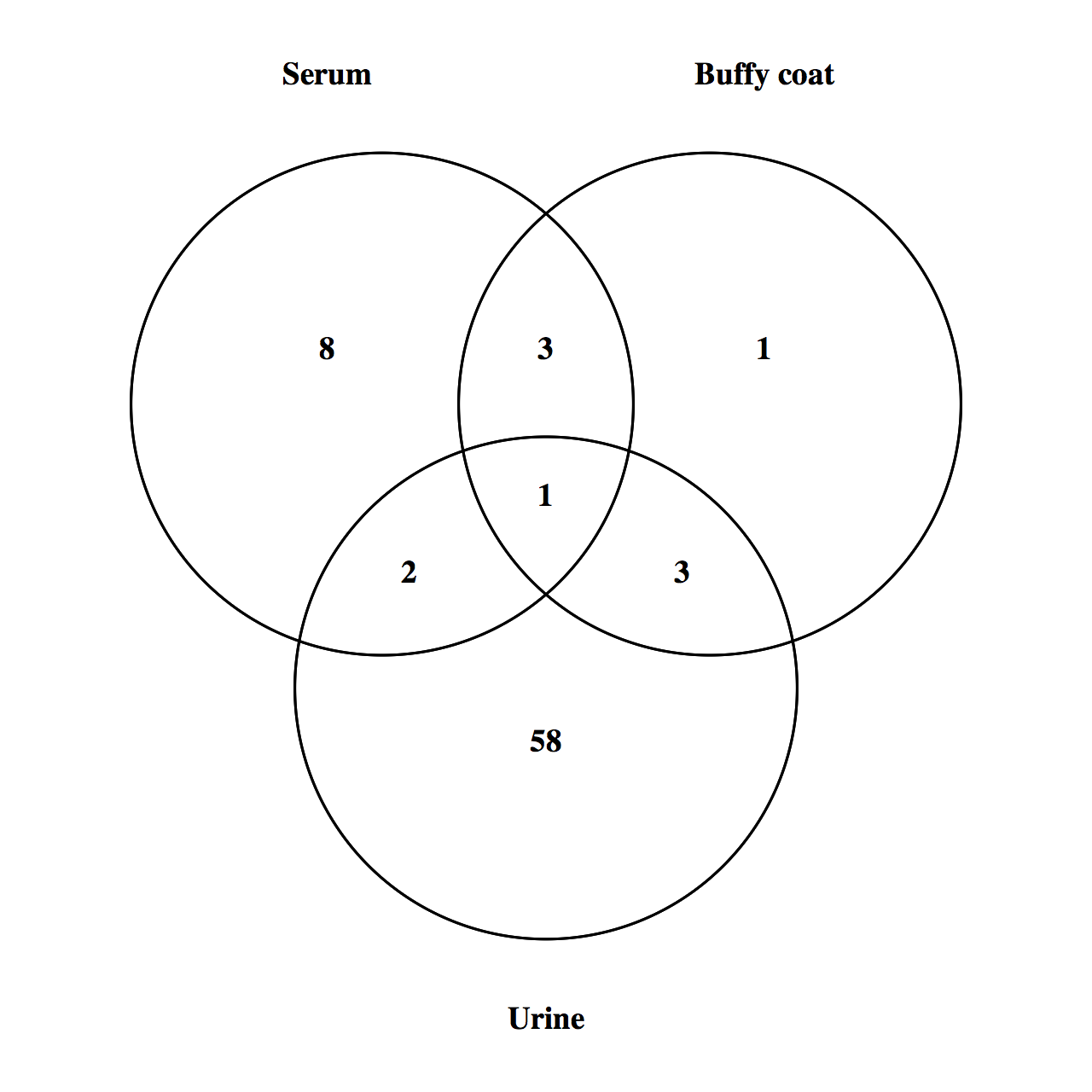


(a) *rrs* qPCR positive (N=76). 3 patients had no urine and 3 no buffy coat sample for testing.


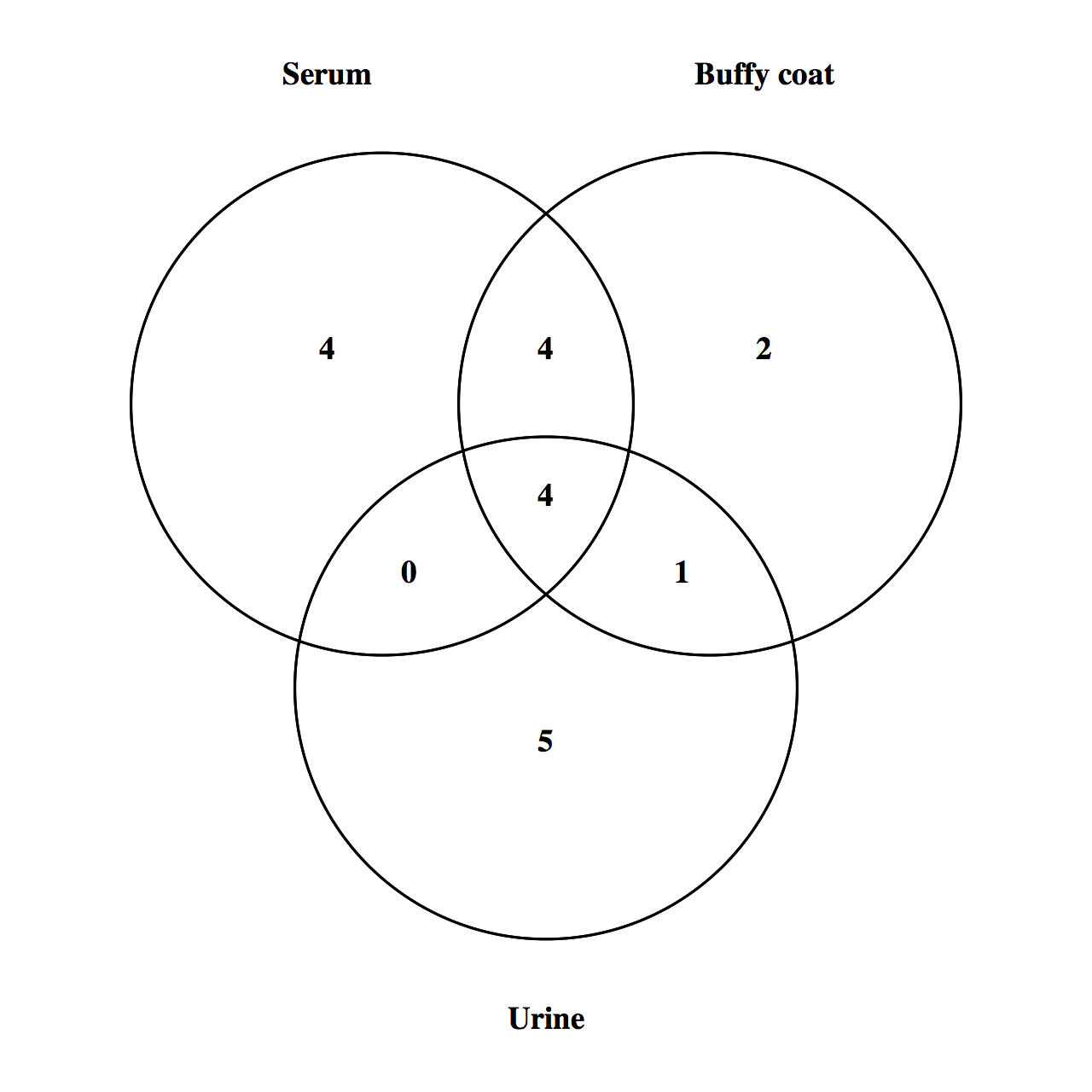


(b) 16SrRNA/*LipL32* qPCR positive for pathogenic *Leptospira* DNA (N=20). 4 patients had no urine and 1 no serum for qPCR testing.
